# High Power, Efficient, and Stable Quantum Dot-Based Downconverters for SWIR Applications

**DOI:** 10.1021/acsphotonics.5c02826

**Published:** 2026-02-06

**Authors:** Aditya Jagadeesh Malla, Katerina Nikolaidou, Miguel Dosil, Mariona Dalmases, Stephy Vincent, Marta Martos Valverde, Gerasimos Konstantatos

**Affiliations:** † ICFO-Institut de Ciències Fotòniques, The Barcelona Institute of Science and Technology, Castelldefels, 08860 Barcelona, Spain; ‡ ICREA-Institució Catalana de Recerca i Estudis Avançats, Passeig Lluís Companys 23, 08010 Barcelona, Spain

**Keywords:** optical downconverters, infrared high power light source, thermal management, energy transfer, colloidal quantum dot

## Abstract

Shortwave infrared light sources are indispensable for various applications, including advanced imaging, spectroscopy, and sensing, yet their widespread adoption is limited by the high cost of epitaxial semiconductors, such as InGaAs. Downconverters (DCs) offer a cost-effective alternative, and quantum dots (QDs) stand out due to their high photoluminescence quantum yield, size-tunable emission, and solution processability. However, QD-DCs suffer from performance degradation under high excitation power densities due to significant heat generation in the process of light absorption. Here we have developed high-power, stable, and spectrally tunable narrowband and broadband SWIR DCs (1000–1600 nm) based on Lead sulfide QDs. By mixing two different-sized QDs, we exploit Förster resonance energy transfer and photon reabsorption to realize a binary system with a high photoluminescence quantum yield of 35%. Embedding the QDs in a poly­(methyl methacrylate) host mitigates local thermal stress on the QDs, enabling standalone DCs with a high emission power density (EmPD) of 110 mW/cm^2^ at 1380 nm. Further optimization with a spectrally selective distributed Bragg reflector for enhanced light extraction and a sapphire substrate for efficient heat dissipation, we achieved a record EmPD of 385 mW/cm^2^ at 1380 nm with optical power conversion efficiency of 10% and operational stability above 230 h at an EmPD of 190 mW/cm^2^. This demonstrates a scalable route to low-cost SWIR light sources, narrowing the performance gap between solution-processed DCs and conventional epitaxial semiconductors.

## Graphical abstract

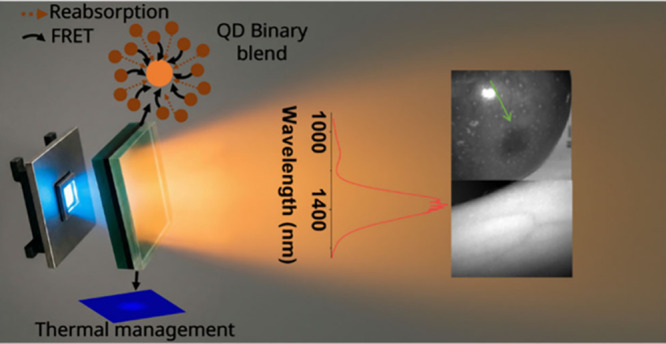

## Introduction

The shortwave infrared (SWIR) spectral region, spanning from 1000 to 1700 nm, has emerged as a critical window for a diverse range of next-generation machine vision technologies, including remote sensing,^1^ nondestructive imaging,^2^ plastic sorting,^3^ automotive,^4^ AR/VR, biomedical imaging,^5^ night vision,^6^ and spectroscopy.^7^ Unlike visible or near-infrared wavelengths, SWIR light is eye safe, suffers less from scattering, has higher atmospheric transmission,^8^ and enables imaging through visually scattering media like smoke^9^ and fog. The moisture absorption in the SWIR region around 1380 nm offers numerous further applications, from monitoring plant growth, food inspection, and imaging tissue moisture to distinguish superficial from deep burns.^10^ Due to the strong water absorption in this band, sunlight is almost completely absorbed, yielding a low-background optical region ideal for remote inspection and surveillance during both day and night. Compact, high-power, and spectrally tailored SWIR light sources are key enablers for expanding these applications into consumer electronics, autonomous systems, robotics, and automotive systems. For such applications to be addressed, a prerequisite for light sources is high emitted power and a high quantum efficiency offered at low cost. However, conventional SWIR-emitting technologies have significant challenges. InGaAs-based light-emitting diodes (LEDs) and laser diodes, though widely used, suffer from high fabrication cost associated with InP wafer scale technology and limited spectral tunability, making them poorly suited for integration into cost-sensitive or large-area platforms requiring high emitted powers. Meanwhile, transition metal-based phosphors,^11–13^ while offering some stability and spectral control, generally produce narrowband emissions, exhibit low absorption cross sections, and require high-temperature processing, hindering their performance in broadband or low-energy excitation systems. Tungsten halogen sources remain the main viable solution for high power delivery in the SWIR, yet are characterized by bulky form factor, lack of modulation (spectral and temporal), very low overall efficiency, and limited lifetime. These drawbacks have driven the search for alternative materials and architectures that can deliver broadband, wavelength-tunable, and thermally stable SWIR emission under practical operating conditions. We posited that in order to develop a close-to-market low-cost scalable SWIR emitting technology, we should capitalize upon established GaAs wafer-scale technology proven to deliver low cost and high power NIR LEDs as the excitation source of colloidal quantum dot downconverters.

Colloidal quantum dots (QDs), particularly lead chalcogenide systems like lead sulfide (PbS)^14–17^ and lead selenide (PbSe),^18,19^ offer a compelling solution. Their solution-processability, size-tunable bandgap, and compatibility with low-temperature fabrication enable flexible integration into various photonic platforms. More importantly, QD-based downconverters (DC)^9,20,21^ absorb visible or NIR photons and re-emit in the SWIR range, providing a simple, low-cost, and scalable way to create customizable SWIR sources using existing GaAs wafer–scale technology, which already enables high-power and low-cost NIR LEDs as excitation. To date, achieving both high optical power output and long-term stability from QD-based SWIR downconverters remains a key bottleneck, especially under high continuous excitation, where thermal degradation can significantly reduce the emission efficiency and operational lifetime.

In this work, we address these limitations by developing high-power, thermally robust SWIR downconverter films based on PbS QDs embedded in a poly­(methyl methacrylate) (PMMA) host and encapsulated between a distributed Bragg reflector (DBR) and a sapphire substrate. This architecture enhances light extraction and reduces thermal stress, achieving a maximum emission power density (EmPD) of 385 mW/cm^2^ at 1380 nm and allowing the use of tunable narrow-band and broadband SWIR emitters. These films exhibit high operational stability under continuous high excitation power density (ExPD). Thermal simulations further validate the role of the DBR-sapphire encapsulation in mitigating local heating, which helps in preserving spectral stability and emission intensity. These results establish a scalable, low-cost pathway toward practical and powerful SWIR light sources based on quantum dots.

## Results and Discussion

### Binary Blend Strategy for Tunable Emission

To achieve highly efficient downconversion, we adopted a binary-blend strategy in which two different-sized QDs were mixed.^11–13^ Larger-bandgap QDs serve as the matrix (m-QD_700_, excitonic peak at 700 nm), while a small fraction (7.5%) of lower-band gap QDs acts as the emitter (e-QD). The emission wavelength could be tuned by selecting e-QD_*x*_, with *x* being excitonic peaks from 1000 to 1600 nm, as shown in Figure 1a,b. Unless specified otherwise, we focus on e-QD_1300_ (emission at ∼1380 nm), referred to as binary blend 1300 (BB-1300). In a typical synthesis, quantum dots (QDs) are capped with oleic acid (OA) to ensure the colloidal stability. Following synthesis, we partially exchange the OA ligands of m-QD_700_ with dodecanethiol (DT).^9,17,24^ The enhanced stability arising from the strong covalent bonding of thiols to PbS QDs is reflected in the higher photoluminescence quantum yield (PLQY) of 65% for DT-capped m-QD_700_ compared to 46% for OA-capped QDs. The transient PL (TRPL) measurements of both OA- and DT-capped m-QD_700_ exhibit monoexponential decay (Figure S1), suggesting a single recombination channel in each case.

**Figure 1:**
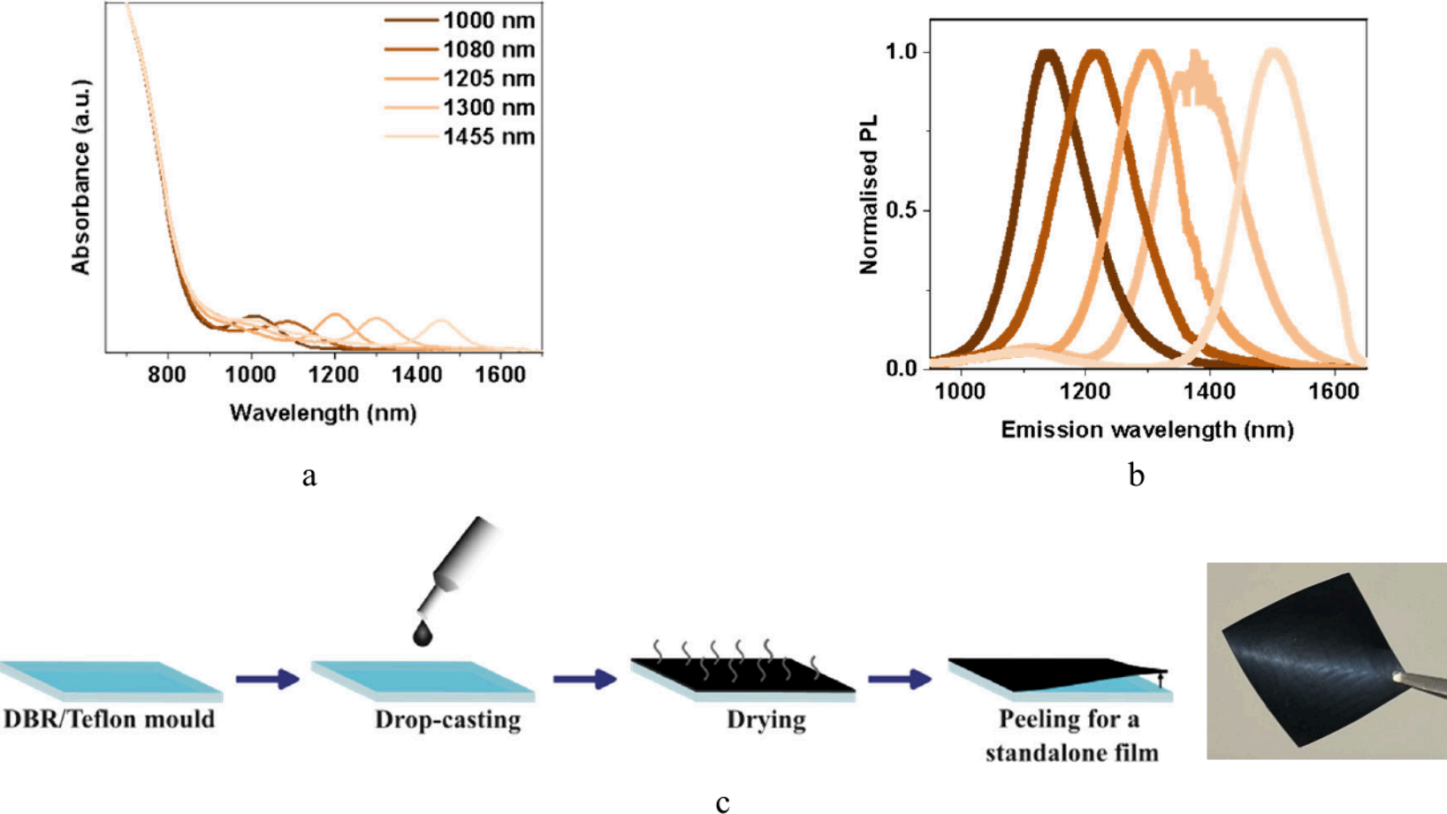
Optical properties and fabrication of standalone DC films. (a) Absorbance spectra of binary blends with different e-QDs with exciton peak spanning from 1000 to 1455 nm, and (b) normalized PL spectra of respective binary blends. (c) A schematic of the fabrication process used to fabricate standalone DC films in a PMMA host and a photograph of a flexible, free-standing film peeled off a mold.

### Design of Polymer-Embedded Quantum Dot Downconverters

The concept of downconverters is based on the absorption of high-energy photons, followed by thermal relaxation of excited carriers to lower-energy states and subsequent radiative recombination that produces lower-energy photons. The thermal relaxation and the nonradiative recombinations generate considerable heat, compromising device performance and stability. To mitigate thermal stress, we spatially dispersed the emitters by embedding PbS QDs in a polymer host. We selected PMMA as the host due to its optical transparency (Figure S2a), processability, and environmental stability. Its negligible absorption in the visible and near-infrared regions minimizes photon losses, while its solution processability enables scalable device fabrication. In addition, PMMA serves as a barrier against oxygen and moisture, improving the device stability. As shown in Figure 1c, the DC films were fabricated by simple drop-casting of QD–PMMA composites, offering cost-effective and scalable production. Transient PL decay measurements (Figure S3a) confirm that embedding QD_700_ in the PMMA matrix does not degrade the PLQY or alter the PL decay lifetimes. These results suggest that the PMMA matrix preserves the QD surface passivation, blocking nonradiative surface defects that could otherwise alter the PL dynamics. This stability is closely linked to the surface chemistry of the QDs, which determines their susceptibility to oxidation. As ligands bind to Pb on the QD surface, the QD_700_ are more resistant to oxidation due to their dominant Lead­(Pb)-rich [111] facets. However, QD_1300_ has [100] facets with exposed sulfur (S) atoms, making them more prone to oxidation.^25^ As seen in Figure S3b, the drop-casted QD_1300_ (without PMMA) film has a significant quenching in the transient PL decay, likely due to enhanced nonradiative defects on the QD surface. Embedding the QD_1300_ in the PMMA host protects the QDs from air and moisture, preventing the degradation of QDs, preserving their solution-phase properties (Figure S3b). The spatial distribution of QDs can be seen in Figure S4. At higher QD loading concentration (10% v/v of 250 mg/mL PMMA to 250 mg/mL QDs), the QDs form small clusters in the PMMA host, resembling the behavior of a QD film rather than isolated QDs in solution, as evidenced in the energy dispersive X-ray (EDX) mapping performed on the cross-section of the DC films, where clusters of QDs are clearly visible.

### Energy Transfer Mechanism in Binary Blends

The absorption and emission spectra of m-QD_700_ and e-QD_1300_ are shown in Figure S5. Films containing only m-QD_700_ exhibited a higher PLQY of 65% compared with 19% for e-QD_1300_. However, in Figure 2a, the steady-state reflection-PL (excitation and collection from the same side of the sample; see schematic in the inset of Figure 2a) of the binary exhibits dominant emission from e-QD_1300_, even though e-QD_1300_ is relatively less efficient and constitutes only 7.5% of the total loading. This suppression of matrix emission suggests energy transfer from m-QD_700_ to the e-QD_1300_. Previous studies^14,22,23^ of binary blends for LEDs relied on short ligands and applied bias to facilitate carrier funnelling from matrix to emitter QDs, suppressing matrix emission. In our case, the long-chain OA and DT ligands hinder direct carrier transport, yet we observe an energy transfer. For energy transfer to occur between the matrix and the emitter QDs, two mechanisms dominate: Förster resonance energy transfer (FRET) and photon reabsorption.

**Figure 2:**
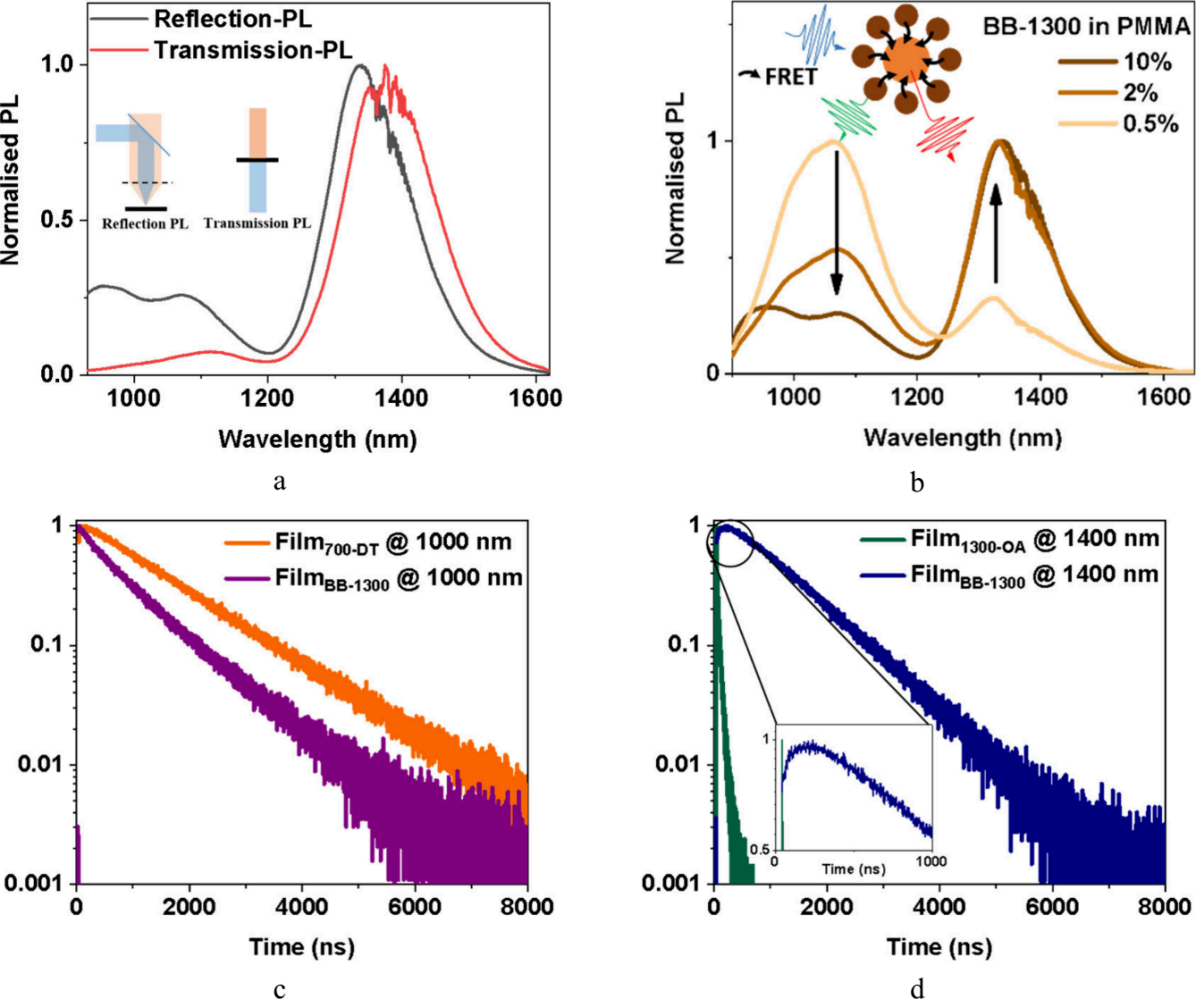
Optical mechanisms at play in BB-1300. (a) The steady state PL of the QD films in the reflection configuration and the transmission configuration. The inset shows the schematic of different methods of PL collection, where the blue indicates excitation, and the orange indicates emission. (b) The steady state PL spectrum in the reflection configuration of different BB-1300 loading concentrations in the PMMA polymer, showing the FRET mechanism from donor (m-QD_700_) to acceptor (e-QD_1300_). (c) Comparison of the transient PL decays of films with m-QD_700_ and BB-1300 at 1000 nm, demonstrating a donor behavior. (d) Comparison of the transient PL decays of films with e-QD_1300_ and BB-1300 at 1400 nm, demonstrating an acceptor behavior.

Because FRET is highly sensitive to donor–acceptor separation,^26^ we fabricated standalone DC films with systematically varied QD loading concentrations in the PMMA matrix to modulate the inter-QD distance, thereby allowing us to validate FRET as the principal energy transfer mechanism. We observed concentration-dependent changes in the relative PL intensities for donor and acceptor QDs in steady-state reflection-PL (Figure 2b). The suppression of the donor emission with increasing the QD loading concentration in the PMMA polymer indicates the FRET mechanism in the binary system.^27^ Figure 2c,d shows spectrally resolved TRPL measurements on QD films comparing individual and blended QDs, revealing the evolution of donor–acceptor roles. Figure 2c shows that at 1000 nm, where emission arises from m-QD_700_, the transient PL decay of BB-1300 (τ = 898 ns) is faster than that of the m-QD_700_ film (τ = 1464 ns), indicating donor-like behavior typical of donor–acceptor systems.^27–29^ A FRET efficiency of 38.7% can be calculated by the following expression:^30^

$$E_{\mathrm{FRET}}=1-\frac{\tau_{\mathrm{DA}}}{\tau_{D}}$$ 1

where τ_DA_ is the PL lifetime of the donor in the presence of the acceptor and τ_D_ is that of the donor alone. At 1400 nm (Figure 2d), where the emission comes from e-QD_1300_, the binary blend shows an initial rise in transient PL, consistent with the acceptor behavior.^27,28^ Figure S6 compares the TRPL decay profiles (monitored at 1400 nm) of BB-1300 embedded in PMMA at two distinct concentrations against those of an e-QD_1300_ reference. At a lower concentration, the decay kinetics of BB-1300 closely mirror those of e-QD_1300_. This similarity suggests that the QDs remain well-dispersed within the PMMA matrix at low loading levels, effectively inhibiting cluster formation and suppressing interparticle FRET dynamics.

We confirm the second energy transfer mechanism of reabsorption from further suppression of the m-QD_700_ emission (Figure 2a) when comparing reflection-PL with the transmission-PL (excitation and collection from either side of the sample – see schematic in the inset of Figure 2a), corresponding to the actual device configuration. We attribute this reduction to the reabsorption of the m-QD_700_ emission by e-QD_1300_. Together, these results show that both mechanisms—FRET and reabsorption—enable energy transfer from m-QD_700_ to e-QD_1300_, leading to dominant emitter QD emission with minimal matrix contribution. Section S7 discusses calculating the contribution of energy transfer via FRET and reabsorption.

### Optical Outcoupling and Thermal Management

Our approach of PMMA-QD composites enables high-performance standalone films that reduce thermal stress on the QDs. The fabricated standalone DC films offer Lambertian emission (Figure S8). The stand-alone film has a maximum optical power conversion efficiency (OPCE) of 7% (Figure S9) and a maximum EmPD of 110 mW/cm^2^ against an ExPD of 2000 mW/cm^2^, beyond which there is a significant drop in performance, as seen in Figure 3b. The PMMA polymer acts as a channel for heat dissipation from the QDs. However, in the standalone film, the heat is accumulated within the polymer itself and becomes a critical bottleneck at elevated ExPD. When the excitation LED is driven at maximum power, the PMMA melts because of the high thermal load (Figure S11). In addition to the thermal limitations, the standalone design inherently presents poor optical outcoupling due to bidirectional emission, which is undesirable in most applications.

**Figure 3:**
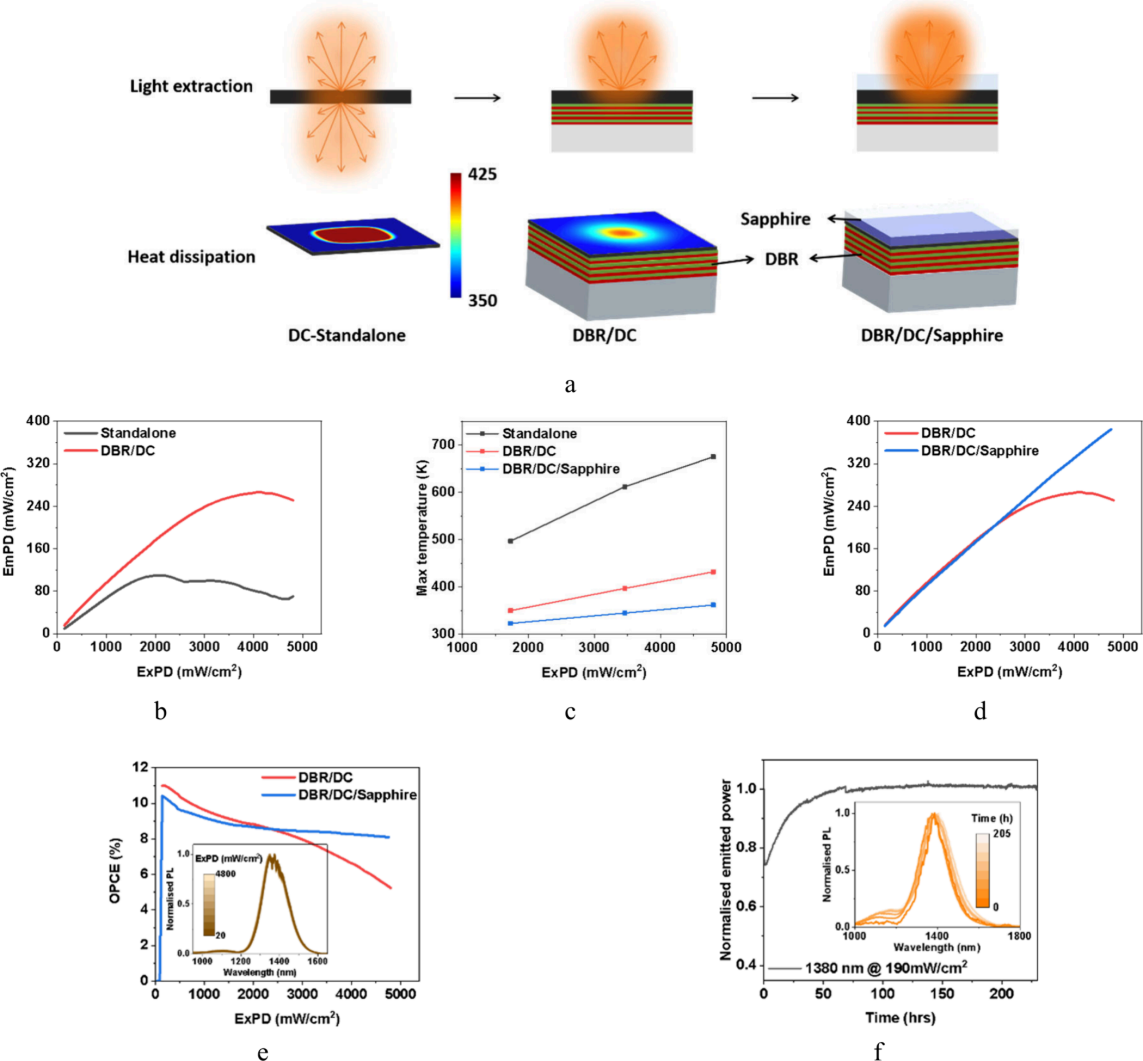
Device engineering with optical and thermal management. (a) Schematic of the device in different scenarios showcasing the light extraction on the top and heat dissipation on the bottom. The surface of the DC shows a simulated heat distribution within the emitter layer. (b) EmPD vs ExPD compares the standalone DC to the DC on DBR, demonstrating the enhanced light out-coupling. (c) Simulated maximum temperatures in DC in different scenarios. (d) EmPD vs ExPD comparing the DBR/DC and DBR/DC/Sapphire structure. (e) OPCE shows a limited efficiency droop for DBR/DC/Sapphire compared to DBR/DC. The inset shows the spectral stability at varying ExPD for the DBR/DC/Sapphire structure. (f) Operational stability of DBR/DC/Sapphire structure emitting 190 mW/cm^2^ at 1380 nm and stable for >230 h with spectral stability shown in the inset.

To maximize the light extraction, we fabricated the DC films on a customized DBR on a glass substrate (Figure S10). The DBR allows the 890 nm excitation light to pass through to the emitter layer while reflecting the longer-wavelength emission, thereby improving light outcoupling and concentrating the emission on one side of the substrate (Figure 3b). In addition to improved light extraction, the DBR structure increases the droop onset from 2000 mW/cm^2^ (standalone film) to 4000 mW/cm^2^, yielding a higher EmPD of 270 mW/cm^2^ (Figure 3b) and a maximum OPCE of 11%. This enhancement is attributed to the DBR on the glass substrate, which not only boosts the optical efficiency but also functions as a heatsink, helping to dissipate heat from the emitter layer. Nevertheless, heat accumulation on the opposite surface still occurs, leading to performance degradation and melting of the emitter layer at high ExPD (see Figure S11).

In order to assess the importance of the heat sink, we performed Finite-element heat transfer simulations of various scenarios using software from Ansys Lumerical. The simulation parameters are discussed in the Supplementary Section S11. As depicted in Figure 3c, the temperature of the standalone film can reach a temperature of 675 K when exposed to intense ExPD. Placing a single heat sink of a glass substrate on one surface of the emitter layer drastically reduced the maximum temperature to 432 K. However, as the heat is still accumulated on the top surface, we simulated a scenario where the emitter layer is sandwiched between a glass and a 600 μm sapphire substrate with thermal conductivity >25 W/mK.^31^ In the final condition of DBR/DC/Sapphire, not only is the temperature further reduced to 362 K (Figure 3c), but also the temperature distribution becomes more uniform, as seen in Figure S11.

We replicated the final simulation condition by adding a single-side-polished sapphire substrate on top of the emitter layer using a diluted PMMA solution as an adhesive layer. This encapsulation of the emitting layer with the DBR and a sapphire substrate eliminates the droop, leading to a maximum OPCE of 10% (Figure 3e), a maximum EmPD of 385 mW/cm^2^ (Figure 3d). It is noteworthy that the maximum emission power is limited by the excitation source. While the DBR/DC/Sapphire architecture exhibits a slightly lower OPCE below 2000 mW/cm^2^, due to optical losses from the sapphire substrate, it excels at higher power densities. Although sapphire is transparent in the SWIR region (Figure S2b), it presents a baseline transmission of 81% (measured as air–sapphire–air). In the actual device, the PMMA adhesion layer (*n* = 1.4) reduces the refractive index contrast, slightly improving transmission (>81%), though a minor drop in OPCE persists. However, beyond 2000 mW/cm^2^, the thermal benefits of the sapphire substrate dominate. To optimize the emission output, we investigated higher e-QD_1300_ loading concentrations within the BB-1300. Increasing the loading to 15% yielded a similar performance to lower concentrations of 7.5% (Figure S12b) but introduced a redshift in the PL spectrum (Figure S12a), attributed to reabsorption. A further increase in the loading proved detrimental, resulting in a notable drop in emitted power density. In order to maximize the absorbed power, we increased the thickness of the emitter layer by stacking multiple layers. However, at higher thickness, strong reabsorption of the emission wavelength is evident from the emission spectrum (Figure S13a), which limits the performance of the DCs (Figure S13b).

The DBR/DC/Sapphire architecture actively dissipates the generated heat from the DC film, offering high performance with minimal droop (Figure 3e) and spectral stability (inset of Figure 3e) throughout the ExPD. This multilayer design not only eliminates the onset of thermal quenching but also improves the long-term operational stability of the device. The DCs with DBR and sapphire were stable for 230 h at an EmPD of 190 mW/cm^2^, as shown in Figure 3f. By mitigating heat accumulation, the OPCE is preserved while also maintaining a stable spectral emission over extended operation times (inset in Figure 3f). Figure 3a illustrates the optical and thermal optimizations required to achieve high-performance DCs.

### Spectral Tunability

To prove the spectral tunability, we fabricated different DCs of varying emission wavelengths (in the SWIR range) with the above DBR/DC/Sapphire architecture, and the performances are shown in Figure S14. The performance of the DCs follows the same trend as the PLQY for the respective binary blends, as shown in Figure S15. Some applications, such as SWIR spectroscopy, require broadband emission. The possibility of tuning the emission wavelength with the size of QDs allows us to engineer the emission spectrum. Broadband emission was realized by two key strategies: multilayer assembly and control of thickness. We layered the downconverters by sequentially stacking the drop-cast films in order, keeping the lower emission energy closer to the excitation source, as shown in Figure S16a. This particular layering of the DCs would allow us two benefits. First, the emitted light from the lower layers has minimal reabsorption from the top layers. Second, the maximum excitation light is absorbed by the lower PLQY films, and there is less absorption for the higher PLQY films (see Figure S15), balancing out the emitted photons and offering a broader SWIR emission with a full-width half-maximum of 407 nm, as shown in Figure S16b. As we mentioned earlier, directly stacking the high-performance narrowband SWIR DCs greatly increases the film thickness and deteriorates the performance due to strong reabsorption. Downconverters were accordingly reoptimized for the purpose of layering for broad emission. We reduced the PMMA-QD solution concentration from 250 mg/mL to 50 mg/mL to have thinner films. This broadband emitter offers a maximum EmPD of 265 mW/cm^2^ (Figure S16c) with a maximum OPCE of 8.6% (Figure S16d). To benchmark our SWIR DCs against existing technologies, we compared their performance metrics (fabrication temperature, emission wavelength, and maximum power) and summarized the comparison in section S16. Our DCs exhibit competitive power output and broad spectral coverage while maintaining low fabrication cost (section S17) and scalability, positioning them as strong candidates for potential SWIR imaging and sensing applications.

### Applications

To demonstrate the versatility of our SWIR DCs, we tested them in several real-world imaging scenarios that highlight their potential in inspection, biomedical, and vision-based applications (in the eye-safe region >1400 nm). When used together with a SWIR camera, DCs enable see-through imaging through common plastic (Figure 4a) materials, which are typically opaque under visible illumination. This capability is crucial for nondestructive inspection and quality control in the packaging and food industries. The downconverters also allowed imaging through double-sided polished undoped silicon (Si) wafers (Figure 4b), demonstrating their utility for semiconductor inspection, where SWIR transparency of Si provides an inherent advantage.

**Figure 4:**
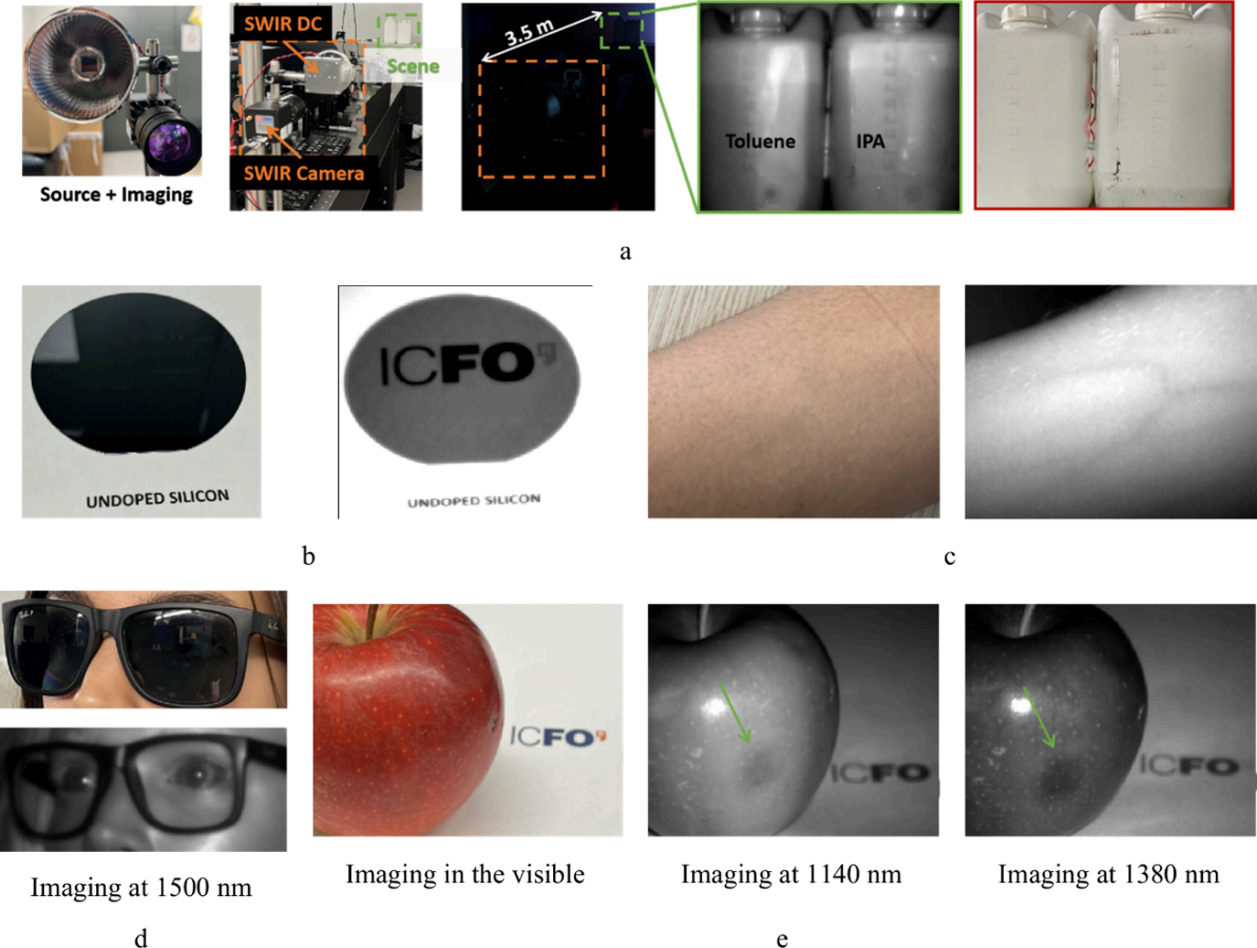
Demonstration of SWIR DCs in real-world applications. Comparison of visible images with SWIR images. All SWIR images were taken in the dark with SWIR DC being the only light source. (a) Night vision and see-through plastic bottles (toluene in the left bottle and isopropanol in the right bottle) placed at 3.5 m away from the light source and the SWIR camera. The orange highlights are the source and camera, whereas the green highlights are the plastic bottles. The SWIR image shows a contrast of the solvents in the bottles, which is clearly absent in the visible image (red highlighted). (b) See-through silicon wafer, demonstrating the strong optical transparency of crystalline Si beyond 1100 nm, enabling inspection of internal wafer structures and defects. (c) Visualization of veins in the arm, exploiting differential absorption of SWIR light by blood and tissue. (d) See-through sunglasses for surveillance, imaged with eye-safe 1500 nm illumination. (e) Defect detection in fruits. On the left is a color photo of the apple, at the middle a SWIR image with 1140 nm illumination, and at the right a SWIR image with 1380 nm illumination, clearly showing the bad spot in the apple. The ICFO logo was obtained from the communications department at ICFO.

SWIR imaging using our DCs was also applied to biological tissues, allowing visualization of veins (Figure 4c) thanks to deeper light penetration and reduced scattering in the SWIR region. Beyond such biomedical applications, in demonstrations relevant to security and surveillance, the SWIR imaging capability also enables see-through observation through tinted sunglasses (Figure 4d), where the scene was illuminated with eye-safe 1500 nm light. Additionally, we demonstrated defect detection on fruit surfaces, such as identifying bad spots on apples, where differences in water absorption and tissue structure provide a natural contrast in SWIR images (Figure 4e). The bad spot is visible with a 1140 nm (peak wavelength) illumination, because the spectrum extends to 1200 nm, which is one of the absorption peaks of water. However, with 1380 nm illumination, where there is a prominent absorption peak of water, the contrast is drastically enhanced. Such contrast is absent in the visible imaging. Together, these demonstrations emphasize the broad applicability of our QD-based SWIR DCs for affordable, compact, and active imaging systems spanning industrial inspection, food safety, surveillance, and biomedical diagnostics.

## Conclusion

We have developed high-power, scalable, solution-processed QD-based SWIR DCs by embedding an engineered mixture of PbS QDs in a PMMA host. This combination offers a 2-fold advantage by making use of FRET mechanisms and light reabsorption to increase optical performance and further allowing a drastic reduction of the thermal stress on the QDs, overall providing EmPD as high as 110 mW/cm^2^. Our approach of embedding QDs in a polymer solution and drop-casting the films offers great versatility and allows the fabrication of films of any shape. The use of a spectrally tuned DBR substrate not only improves output directionality but also works as a heat sink, improving the performance of the DCs. By adding an extra sapphire substrate on top, we created a DBR/DC/sapphire structure that further helps dissipate the heat from the lateral surfaces of the emitter film, thereby increasing the thermal stability of the DCs and enabling a record EmPD of 385 mW/cm^2^ with an OPCE of 10% with minimal droop. We demonstrated spectrally tunable high-power DCs by varying the e-QDs. By strategically stacking different narrowband DCs, we displayed a broadband DC emission with a fwhm greater than 407 nm, covering a significant part of the SWIR region. These results, along with real-world application demonstrations, prove solution-processed QD-based DCs as a promising and cost-effective route toward high-power, spectrally tunable, and stable SWIR light sources. Beyond bridging the performance gap with epitaxially grown semiconductors, our approach paves the way toward scalable integration of SWIR emitters in next-generation imaging, sensing, and communication technologies.

## Experimental Section

### Synthesis of PbS Quantum Dots

*1300 nm excitonic peak PbS QDs*: PbS QDs were synthesized under an inert atmosphere by the hot injection method. Briefly, 0.446 g of PbO, 50 mL of 1-octadecene (ODE) and 3.8 mL of oleic acid (OA) were heated at 100 °C under vacuum for 1h to form the lead precursor (lead oleate). Once under argon, a solution of 90 μL of hexamethyldisilathiane (HMS) in 3 mL of ODE was quickly injected. After 6 min of reaction, a second solution of 75 μL of HMS in 9 mL of the ODE was injected dropwise. Subsequently, the solution was cooled naturally to room temperature. PbS QDs were precipitated with the addition of a mixture of acetone/ethanol and redispersed in anhydrous toluene. This purification process was repeated two more times. Finally, the concentration of PbS QDs was adjusted to 250 mg/mL. The last step of purification and the adjustment of the concentration were done inside the glovebox in order to avoid oxidation. 1200 and 1455 nm excitonic peak PbS QDs were synthesized identically, varying the HMS amounts: 110 and 75 μL were used in the first injection, respectively, and 50 and 100 μL in the second.

*700 nm excitonic peak PbS QDs*: 0.892 g of PbO, 36 mL of ODE and 3.2 mL of oleic acid were degassed for 1 h under vacuum at 100 °C. Once under argon, the temperature was set at 80 °C, and 420 μL of HMS in 5 mL of ODE was quickly injected. After 15 s, the reaction was quenched with cold acetone, and QDs were collected by precipitation. The PbS QDs were purified by dispersion/precipitation with anhydrous toluene and a mixture of acetone/EtOH three times. Finally, the concentration was adjusted to 50 mg/mL, and the sample was stored at a low temperature to avoid Ostwald ripening. PbS QDs with 1000 and 1080 nm excitonic peaks were synthesized following a similar procedure, increasing the reaction temperature to 95 °C and adjusting the oleic acid volume to 5 and 6.4 mL, respectively. After precursor injection, both reactions were allowed to cool down naturally to ambient temperature.

*Ligand exchange process*: The ligand exchange process to exchange part of the oleic acid bound to the surface of the QDs to 1-dodecanethiol (DT) was performed in the liquid phase. For a solution of PbS QDs (of 50 mg/mL) containing 100 mg of PbS QDs, 0.05 mmol of DT was added, and the solution was stirred vigorously for 30 min. Subsequently, the sample was washed two times with acetonitrile and redispersed with anhydrous toluene. Finally, the concentration was adjusted to 250 mg/mL.

### Fabrication of the Downconverter Films

In order to fabricate the downconverters, we first dissolve PMMA (MW = 120 kDa) powder in chloroform at a concentration of 250 mg/mL. In parallel, we also prepare a highly concentrated quantum dot solution at 250 mg/mL in toluene. The quantum dot solution is then added to the PMMA solution at 10% in volume. The highly viscous PMMA QD mixture is then drop-cast onto a desired substrate. Drop-casting onto a Teflon mold and peeling off after drying gives a standalone film. This technique also allows the fabrication of films in any shape, depending on the mold. In the case of a DC-sandwich, to place the sapphire on top of the film, we used 100 mg/mL of PMMA solution as an adhesion layer between the downconverter and the sapphire on top.

### Characterization Methods

*Photoluminescence*: Steady-state photoluminescence of the films was recorded by using a 1D-InGaAs array (iDus 1.7, Oxford Instruments) coupled to a 500 mm monochromator (Shamrock i500, Oxford Instruments). PL was collected in two different configurations. The so-called transmission-mode PL was recorded by exciting the samples from the back and collecting light from the other side of the film. For the case of the reflection mode, PL was recorded from the same side as the excitation.

*Time-resolved photoluminescence*: TRPL measurements were obtained by exciting the films with an 827 nm laser diode (LDH-D-C-830, PicoQuant) and recording the traces with a single-channel superconducting nanowire single photon detector (ID281, IDQuantique). Photon events were time-tagged using a time-correlated single photon counting module (ID1000, IDQuantique) and the intensity-time histograms were postprocessed with a custom Python 3 script.

*PLQY*: The DC films were measured inside an integrating sphere (QuantaPhi, Horiba Scientific) coupled to the aforementioned spectrograph (Shamrock i500, Oxford Instruments). Absorption at the excitation wavelength and the PLQY were measured by following the methods described by De Mello et al.^32^

*Scanning electron microscopy and energy-dispersive X-ray*: SEM-EDX characterization was performed using a Zeiss GeminiSEM 560 scanning electron microscope equipped with Oxford Ultim Max Infinity (UM40) EDX detector (40 mm^2^ sensor size).

*Downconverter performance*: The excitation source is an LED with an emission peak at 890 nm (SMB1N-890DS) purchased from RoithnerLaserTechnik GmbH. Films were placed at a known distance from the Newport 818-IR photodetector. Under the assumption of a Lambertian emission profile, the total emitted power can be determined based on the solid angle of the detector. Optical filters were used to block the excitation light.

## Supplementary Material



## Notes

TRPL plot of QDs with different ligands, transmission spectra of PMMA and sapphire, TRPL of QDs in different environments, SEM and EDX images of DCs, absorption and emission spectra of QDs, TRPL plots of binary blends at different concentrations, the calculations for FRET and reabsorption contributions, DC emission profile, performance of standalone DC, reflection spectrum of DBR, the simulation parameter and simulated surface profiles with photographs of real devices, performance of DCs with varying emitter QD loading concentration, film thickness, spectral tunability (with PLQY plot), and broadband emission. Comparison of our developed DC with current technologies and commercial LEDs, and pricing of our DCs (PDF)
