# Supplementary material for: High Power, Efficient, and Stable Quantum Dot-Based Downconverters for SWIR Applications
Source: ACS Photonics. 2026 Feb 6;13(4):1158–66. doi: 10.1021/acsphotonics.5c02826 (PMC12922171; doi:10.1021/acsphotonics.5c02826)
Supplement: Supplementary file 1 [file ph5c02826_si_001.pdf]

Supporting information

High Power, Efficient and Stable Quantum Dot-based Downconverters for SWIR Applications

*Aditya Jagadeesh Malla[1], Katerina Nikolaidou[1], Miguel Dosil[1], Mariona Dalmases[1], Stephy Vincent[1], Marta Martos Valverde[1], Gerasimos Konstantatos[1,2]**

[1]ICFO-Institut de Ciències Fotòniques, The Barcelona Institute of Science and Technology, Castelldefels, 08860 Barcelona, Spain.
[2]ICREA-Institució Catalana de Recerca i Estudis Avançats, Passeig Lluís Companys 23, 08010 Barcelona, Spain.
*e-mail: gerasimos.konstantatos@icfo.eu

S1: Effect of dodecanethiol on the matrix QDs

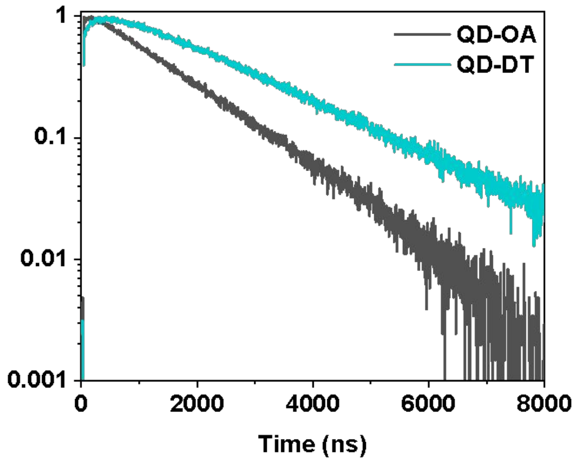

Figure S1: Comparing the transient PL of the m-QD700 with both oleic acid and dodecanethiol, exhibiting mono-exponential PL decay.

S2: Optical transmission of PMMA film and sapphire substrtae

A particular PMMA film mimicking the downconverters (DCs) was prepared without any QDs to measure the transmission spectrum with an integrating sphere.

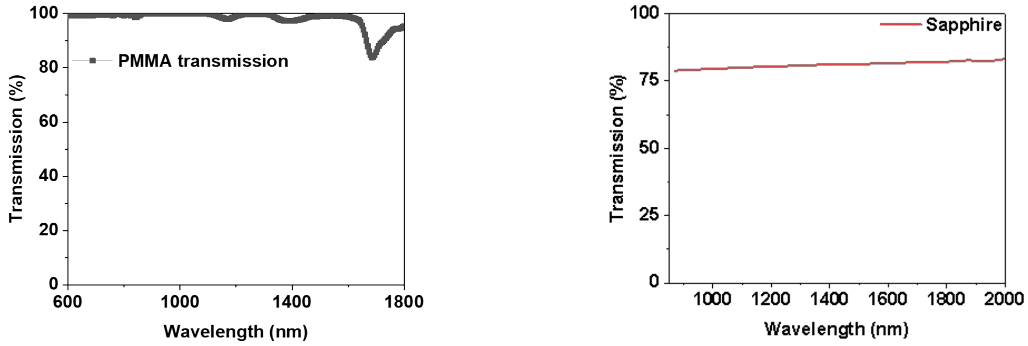

a b

Figure S2: Optical transmission spectrum of a **a** PMMA film and **b** sapphire substrate.

S3: Effect of PMMA on optical properties

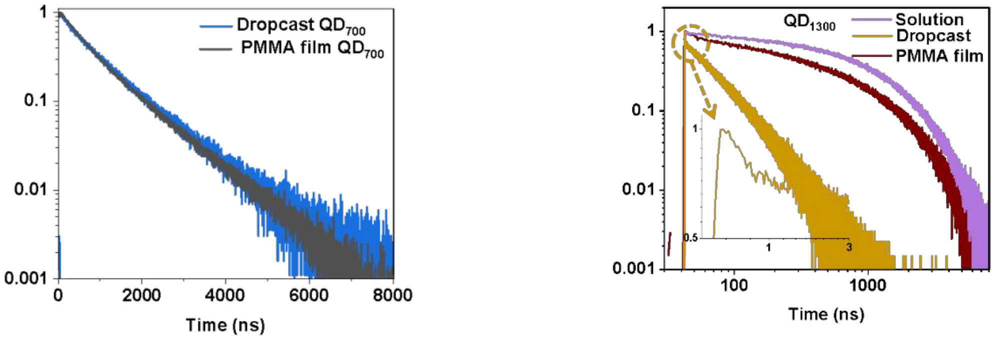

a b

Figure S3: Transient PL decay of **a** QD_{700} comparing the dropcast film to the PMMA film and **b** QD_{1300} comparing the solution to the dropcast film and the PMMA film. The inset in **b** is a close-up of the transient PL decay for dropcast QD_{1300}.

S4: SEM images of DC films

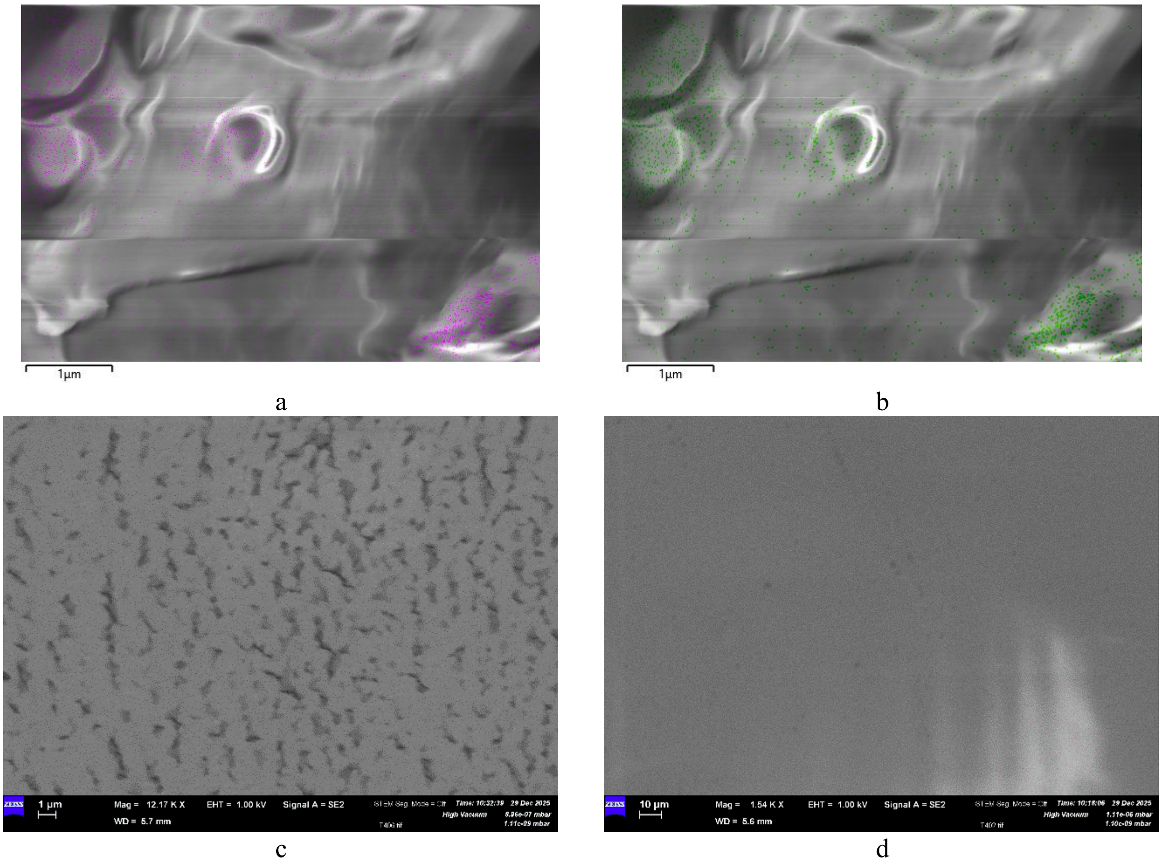

Figure S4: SEM and EDX analysis of DC films. Cross-sectional EDX elemental maps showing the distribution of **a** Lead (Pb) and **b** Sulfur (S). The spatial correlation between Pb and S signals indicates cluster formation within the film. **c,d** are the surface SEM images of the DC films at different scales. The bright streaks in (d) are attributed to surface charging of the PMMA film matrix.

S5: Optical characteristics of individual QDs

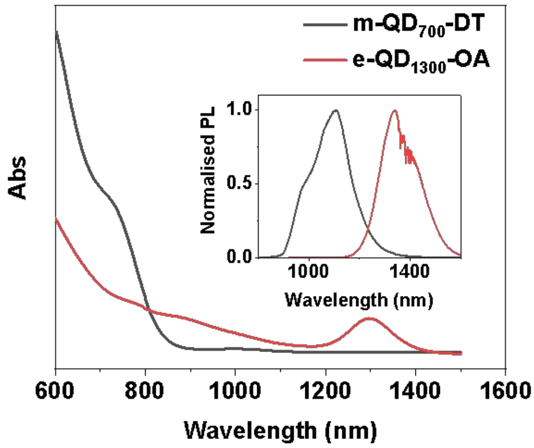

Figure S5: The optical properties: absorption and emission (inset) of m-QD_{700} and e-QD_{1300} individually before mixing.

The noise around 1400 nm is due to strong water absorption from the atmosphere.

S6: TRPL of binary blend QD loading concentration

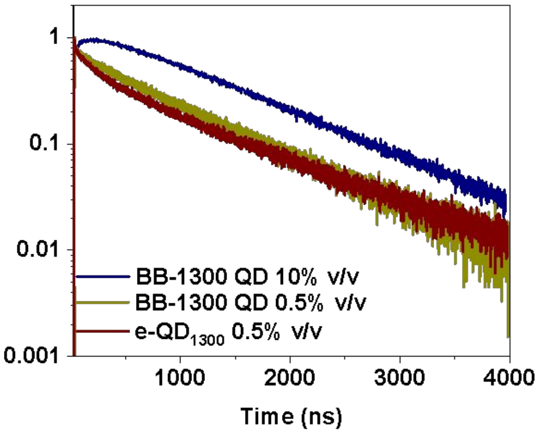

Figure S6: TRPL decay characteristics (at 1400 nm emission) of varying BB-1300 QDs loading concentration in PMMA compared with e-QD_{1300} in PMMA

S7: FRET and reabsorption contributions

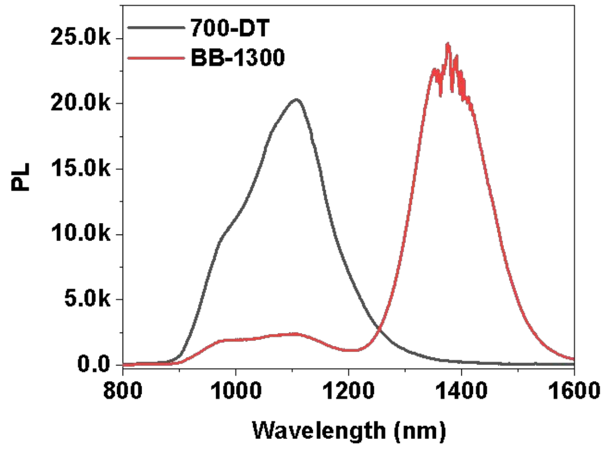

Figure S7: The PL spectrum comparing the emission from m-QD_{700} in the presence and absence of the acceptor e-QD_{1300}.

We can calculate the total energy transfer by:

$$ET = 1 - \frac{I_{DA}}{I_D}$$

where I_D and I_{DA} are the integrated intensity of the emission from m-QD_{700} in the absence and presence of the acceptor. We calculate ET to be 0.883. Assuming the PLQY of m-QD_{700} is unaffected in the presence of acceptor, and given the FRET efficiency of 0.387, we can calculate the contribution of reabsorption (0.496) by,

$$ET_{reabsorption} = ET_{total} - ET_{FRET}$$

S8: Emission profile of the DC films

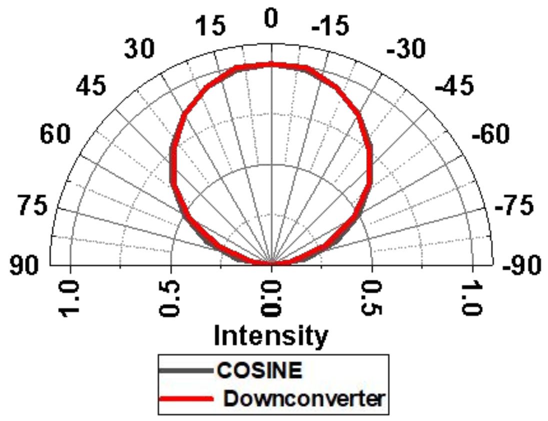

Figure S8: Emission profile of a planar DC compared with the Lambertian profile, where the intensity follows the cosine of the angle.

S9: Standalone DC performances

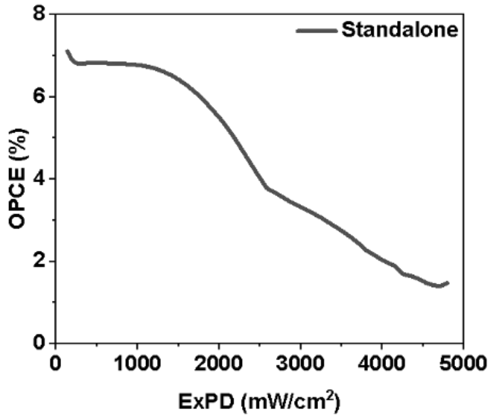

Figure S9: OPCE against ExPD for the standalone DC emitting at 1380 nm.

S10: Spectral properties of the DBR substrate

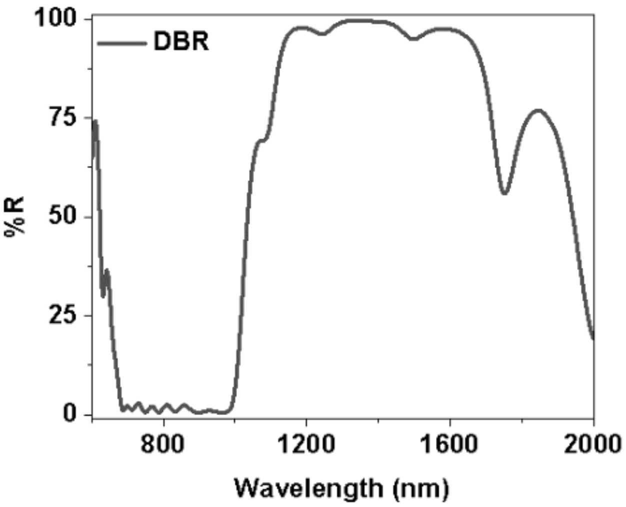

Figure S10: Reflection of the DBR substrate.

S11: Finite-element heat transfer simulations

We performed Lumerical Heat simulations at various power densities for different scenarios.

Parameters considered for the simulation:

Total power for heat is based on the formula:

$$P_{heat} = P_{abs} - P_{emi} \quad (1)$$

Considering $P_{abs} = N_{abs}E_{abs},\ P_{emi} = N_{emi}E_{emi},\ \eta = \frac{N_{emi}}{N_{abs}}$

$$P_{heat} = P_{abs} - N_{emi}E_{emi} \quad (2)$$

$$P_{heat} = P_{abs} - \eta\, N_{abs}E_{emi} \quad (3)$$

$$P_{heat} = P_{abs} - \eta \frac{P_{abs}}{E_{abs}} E_{emi} \quad (4)$$

$$P_{heat} = P_{abs}\left(1 - \eta \frac{E_{emi}}{E_{abs}}\right) \quad (5)$$

Hence, we consider the Stokes shift in the system, and there is heat generation even if the PLQY (η) is 100%.

$P_{abs} = 0.7 \times P_{excitation}$, $\eta = 0.3$, Heat convection = 10 W/m^2K, Emissivity = 0.9

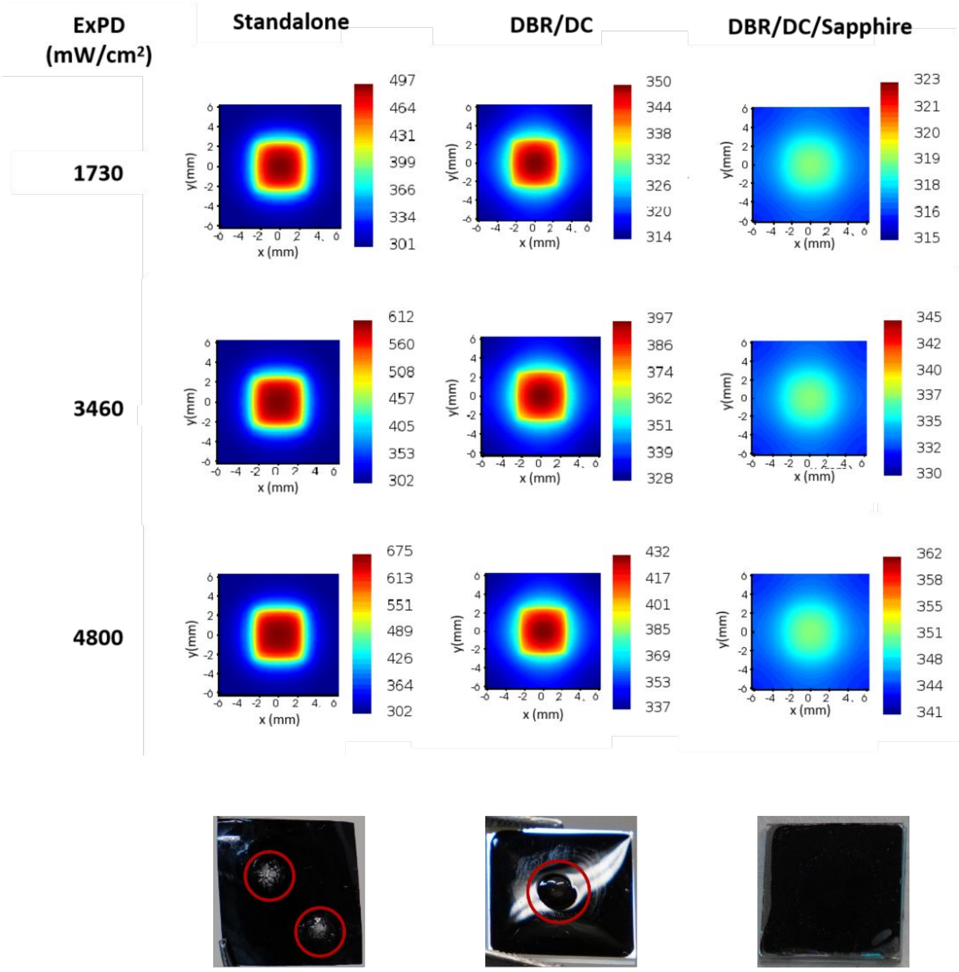

Figure S11: Simulated 2D surface profiles with temperature for different architectures with varying ExPDs. At the bottom are the photographs of the respective devices after the measurements. The standalone and DBR/DC devices have burning of the emitter layer due to extreme heat.

S12: e-QD1300 loading

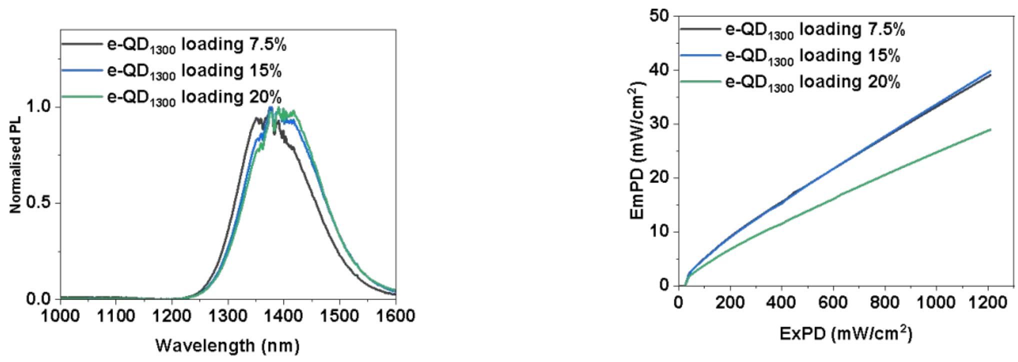

Figure S12: **a** PL and **b** EmPD of SWIR DBR/DC/sapphire with varying e-QD loading into the BB-1300.

S13: Emission layer thickness

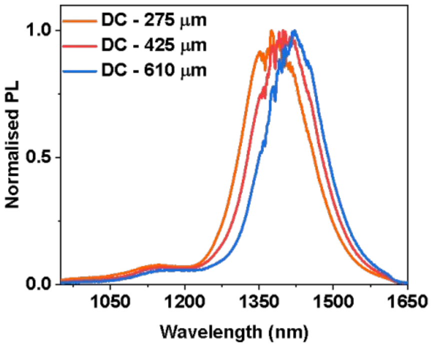

a

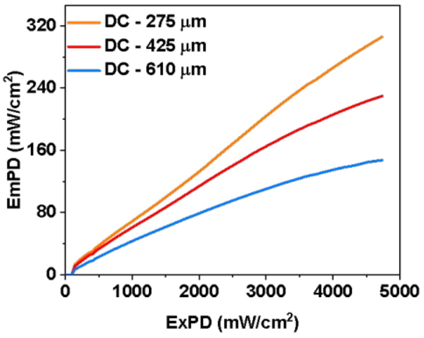

b

Figure S13: **a** PL and **b** EmPD of SWIR DBR/DC/sapphire with varying thickness

S14: Spectral tunability:

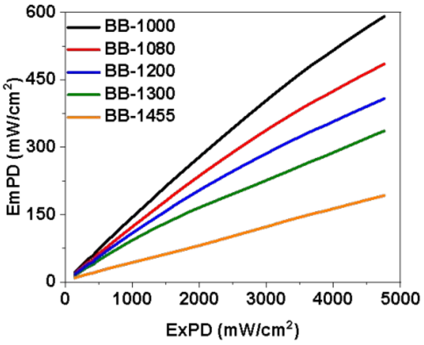

a

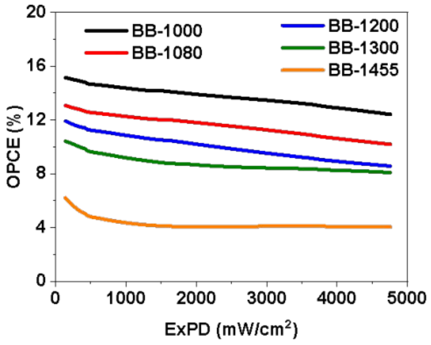

b

Figure S14: **a** EmPD and **b** OPCE against ExPD for varying wavelengths with DBR/DC/Sapphire architecture

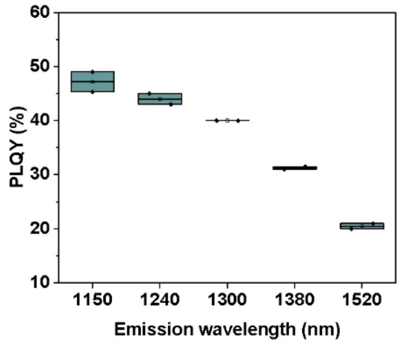

Figure S15: PLQY of the SWIR DCs with different emission wavelengths.

S15: Broadband emission:

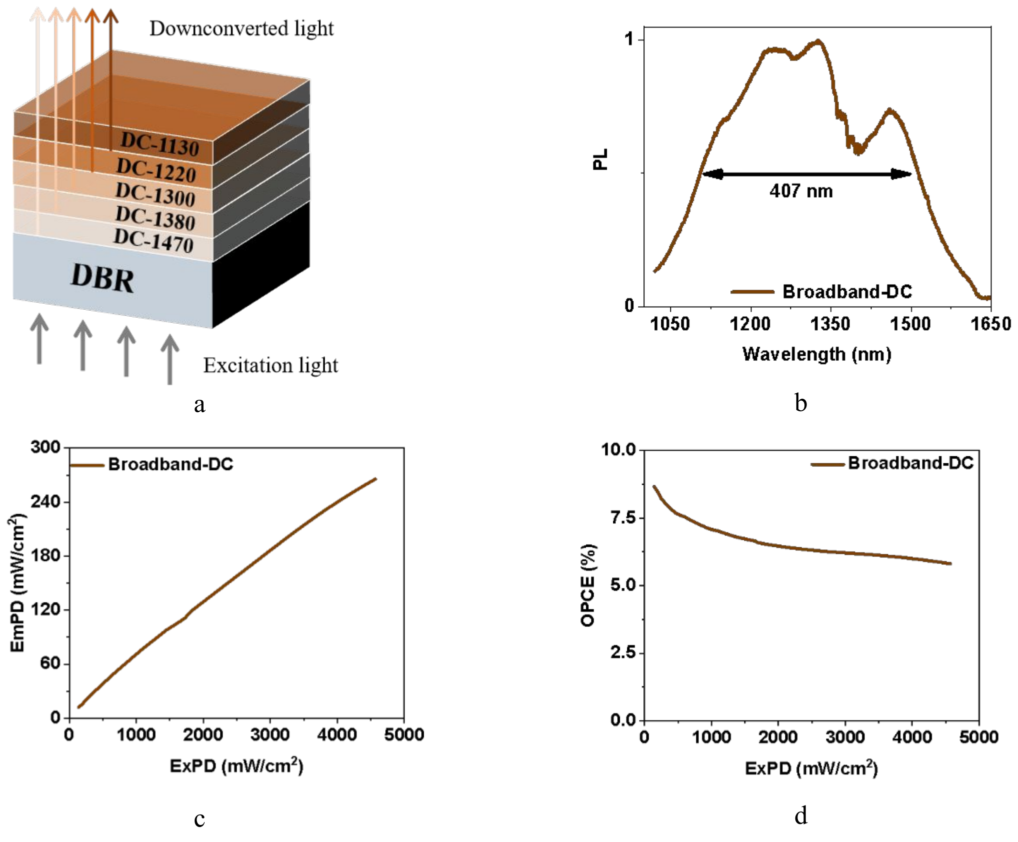

sss

Figure S16: **a** Device structure (stacking), **b** emission spectrum, **c** EmPD vs ExPD, and d OPCE of broadband SWIR DC.

S16 Comparison with other notable works

Emitter	Fab. Temp (°C)	λ (nm)	Maximum Power	Reference
10 mm long Yb,Er:Glass luminescent concentrator		1550	850 mW	1
$LiMgPO_4:Cr^{3+},Ni^{2+}$	1000	1380	2.7 mW	2
$MgGa_2O_4: Cr^{3+},Ni^{2+}$	1350	1260	14.9 mW	3
$LiScGeO_4$	1150	1120	4.78 mW	4
YAGG-Ni-Zr-H_3BO_3	1500	1450	1.25 mW	5
$(Li/Mg)O:Ni^{2+}$	1400	1335	19.7 mW	6
$MgO:Cr^{3+},Ni^{2+}$	1450	1335	27.4 mW	7
PbS QDs	RT	1050-1650	14 mW	8
PbS QD/LLDPE composite	RT	1517.9	0.466 mW/cm^2	9
PbS QDs in cellulose	RT	1380	54 mW/cm^2	10
PbSe QDs	RT	950<br>1550<br>1960	1.09 mW<br>0.69 mW<br>0.16 mW	11
Commercial LEDs from Roithner Laser Technik GmbH (InGaAsP)		1100<br>1200<br>1300<br>1450<br>1550	280 mW<br>180 mW<br>160 mW<br>72 mW<br>50 mW	12
Our work	RT	1140<br>1215<br>1300<br>1380<br>1500	153 mW<br>126 mW<br>106 mW<br>100 mW<br>50 mW	

Table S1: Summary of performance of our SWIR DCs compared with previously reported systems and commercial LEDs.

S17: Pricing of SWIR DCs to InGaAs LEDs:

SWIR DC		Excitation source	
Component	Purchase price (€)	Component	Purchase price (€)
DBR	0.5	Excitation LED (purchase price, low volume)	13.65
Emitter	0.3	PCB&casing (purchase price, low volume)	6.5
Sapphire	0.5		
Total	1.3	Total	20.15

Assuming a 100 mW aimed optical power at s1300 nm or 50 mW at 1550 nm, a commercial SWIR LED is priced at €43[13], whereas the SWIR DCs are priced at €21.5, which can be substantially reduced further under large-scale volume production.

References:

(1) Pichon, P.; Blanchot, J.-P.; Balembois, F.; Georges, P. New LED-Based High-Brightness Incoherent Light Source in the SWIR. *Opt. Express* **2018**, *26* (7), 9353. https://doi.org/10.1364/OE.26.009353.

(2) Miao, S.; Liang, Y.; Zhang, Y.; Chen, D.; Wang, X.-J. Blue LED-Pumped Broadband Short-Wave Infrared Emitter Based on LiMgPO4:Cr3+,Ni2+ Phosphor. *Adv. Mater. Technol.* **2022**, *7* (11), 2200320. https://doi.org/10.1002/admt.202200320.

(3) Miao, S.; Liang, Y.; Shi, R.; Wang, W.; Li, Y.; Wang, X.-J. Broadband Short-Wave Infrared-Emitting MgGa2O4:Cr3+, Ni2+ Phosphor with Near-Unity Internal Quantum Efficiency and High Thermal Stability for Light-Emitting Diode Applications. *ACS Appl. Mater. Interfaces* **2023**, *15* (27), 32580–32588. https://doi.org/10.1021/acsami.3c05980.

(4) Miao, S.; Liang, Y.; Zhang, Y.; Chen, D.; Wang, X.-J. Broadband Short-Wave Infrared Light-Emitting Diodes Based on Cr3+-Doped LiScGeO4 Phosphor. *ACS Appl. Mater. Interfaces* **2021**, *13* (30), 36011–36019. https://doi.org/10.1021/acsami.1c10490.

(5) Yuan, L.; Jin, Y.; Wu, H.; Deng, K.; Qu, B.; Chen, L.; Hu, Y.; Liu, R. S. Ni2+-Doped Garnet Solid-Solution Phosphor-Converted Broadband Shortwave Infrared Light-Emitting Diodes toward Spectroscopy Application. *ACS Appl. Mater. Interfaces* **2022**, *14* (3), 4265–4275. https://doi.org/10.1021/ACSAMI.1C20084/ASSET/IMAGES/LARGE/AM1C20084_0008.JPEG.

(6) Liu, B.-M.; Gu, S.-M.; Huang, L.; Zhou, R.-F.; Zhou, Z.; Ma, C.-G.; Zou, R.; Wang, J. Ultra-Broadband and High-Efficiency Phosphors to Brighten NIR-II Light Source Applications. *Cell Rep. Phys. Sci.* **2022**, *3* (10). https://doi.org/10.1016/j.xcrp.2022.101078.

(7) Liu, B.-M.; Guo, X.-X.; Cao, L.-Y.; Huang, L.; Zou, R.; Zhou, Z.; Wang, J. A High-Efficiency Blue-LED-Excitable NIR-II-Emitting MgO:Cr3+,Ni2+ Phosphor for Future

Broadband Light Source toward Multifunctional NIR Spectroscopy Applications. *Chem. Eng. J.* **2023**, *452*, 139313. https://doi.org/10.1016/j.cej.2022.139313.
(8) Pradhan, S.; Dalmases, M.; Konstantatos, G. Solid-State Thin-Film Broadband Short-Wave Infrared Light Emitters. *Adv. Mater.* **2020**, *32* (45). https://doi.org/10.1002/adma.202003830.
(9) Dong, H.; Xuan, T.; Xie, R.-J. Lead Sulfide Quantum Dot-Linear Low-Density Polyethylene Composites for near-Infrared Mini-LEDs. *J. Lumin.* **2024**, *273*, 120707. https://doi.org/10.1016/j.jlumin.2024.120707.
(10) Vincent, S.; Nikolaidou, K.; Dalmases, M.; Dosil, M.; Malla, A.; Wang, Y.; Konstantatos, G. Flexible Large Area SWIR Colloidal Quantum Dot Down Converters Based on Scalable Manufacturing Processes. *Adv. Mater. Technol.* **2025**, *10* (12), 2401960. https://doi.org/10.1002/admt.202401960.
(11) Yan, L.; Shen, X.; Zhang, Y.; Zhang, T.; Zhang, X.; Feng, Y.; Yin, J.; Zhao, J.; Yu, W. W. Near-Infrared Light Emitting Diodes Using PbSe Quantum Dots. *RSC Adv.* **2015**, *5* (67), 54109–54114. https://doi.org/10.1039/C5RA08130K.
(12) *Roithner Lasertechnik - High Power Single Chip LEDs, SMD LEDs*. https://www.roithner-laser.com/led_highsingle_smd.html (accessed 2025-11-03).
(13) *Roithner Lasertechnik - High Power Single Chip LEDs, SMD LEDs price list*. https://www.roithner-laser.com/pricelist.pdf (accessed 2025-11-03).
